# Youth experiences with and perspectives on long covid

**DOI:** 10.1186/s12889-023-16899-8

**Published:** 2023-10-20

**Authors:** Sarosh Irani, Claire Chang, Leigh Morrison, Marika Waselewski, Tammy Chang

**Affiliations:** 1 University of Michigan Medical School, University of Michigan, Ann Arbor, MI USA; 2 Department of Family Medicine, University of Michigan, 2800 Plymouth Rd Bldg 14, Room G128, Ann Arbor, MI 48109 USA; 3 Institute for Healthcare Policy and Innovation, University of Michigan, Ann Arbor, MI USA

**Keywords:** Long covid, Mental health, COVID-19, Youth health

## Abstract

**Background:**

Research on the long-term effects of COVID-19 infection is ongoing, and the psychological and physical impacts of Long Covid on youth is poorly understood. To assess these impacts, we surveyed youth regarding their experiences with, and perspectives on, the long-term effects of COVID-19.

**Methods:**

We conducted a nationwide text message survey of youth ages 14–24 years in the United States. The survey asked four open ended questions regarding their experiences and perceptions regarding the long-term effects of COVID-19. Qualitative data was analyzed independently by three investigators using thematic analysis. Prevalence of codes were summarized using descriptive statistics.

**Results:**

Among 1150 participants, 991 responded to at least one survey question (response rate 86.1%). The vast majority of our sample had COVID-19 or knew someone who did (75%), and approximately one third (32%) of youth indicated that they knew someone who had experienced symptoms consistent with Long Covid. Many youth (50%) reported worry and concern about Long Covid even if they, or someone they knew, did not have Long Covid. Among youth who were not concerned about Long Covid, the most commonly reported reasons were having received the vaccine (29%) and not having a prior COVID-19 infection (24%).

**Conclusions:**

Our findings suggest that among younger populations, there is significant concern regarding the long-term effects of COVID-19. Vaccination campaigns and youth-centered public health communication about Long Covid may not only reduce COVID-19 transmission, but also alleviate worries and concerns about Long Covid among youth.

## Introduction

Over 50 million Americans have been diagnosed with COVID-19 caused by SARSCoV-2 since 2020 [1], and there is increasing evidence that some patients experience a significant ongoing symptom burden from the disease [2–4]. Patients created the name “Long Covid” in 2020 to describe their experience of persistent symptoms [5]. Also called “post-COVID syndrome” or “post acute sequelae of SARS-CoV-2 (PASC),” these persistent symptoms affect the sensory, neurologic, and cardiorespiratory systems, as well as mental health [6–8]. Though the definition of Long Covid lacks consensus [9], the term typically refers to symptoms that are present more than 3–4 weeks after diagnosis, a time period considered the acute phase of COVID-19 [10, 11]. One study found that nearly 30% of non-hospitalized COVID-19 patients were still reporting symptoms 2 months after infection [12]. Another sample of 2000 non-hospitalized and hospitalized patients found less than 1% of patients indicated that they were symptom-free 80 days after infection [13].

Among youth, data on the prevalence and major features of Long Covid is variable [14]. The prevalence of Long Covid in youth has been reported anywhere from 4 to 66%. There is evidence that youth who have Long Covid have high rates of fatigue, mood symptoms, and impaired sleep [15]. In addition, several studies have demonstrated high rates of multisystem inflammatory syndrome in children (MIS-C) post-COVID, among other conditions [16, 17]. At the same time, few existing studies of Long Covid among youth have examined the emotional and psychological impacts of Long Covid on this population. Youth who have not personally experienced Long Covid symptoms are likely to still experience consequences of Long Covid, especially if individuals in their families and communities are affected.

Neurobiological changes during adolescence result in heightened emotional reactivity, and during adolescence individuals are at especially high risk for developing psychiatric disorders such as anxiety and depression [18, 19]. The negative impact of the COVID-19 pandemic on the mental health of youth has been described [20, 21]. Studies show that youth now suffer from higher rates of depressive and anxious symptoms as compared to pre-pandemic estimates, specifically with high levels of fear and concern regarding the impact of COVID-19 on their lives [21]. Factors associated with anxiety among youth range from fear of oneself or loved ones contracting COVID-19, to concern about the economic and social repercussions of the pandemic [22]. Furthermore, the self-reported general mental health of youth has declined compared to pre-pandemic times [23–25], and youth have had increased rates of suicidal ideation during the pandemic [26–28]. Even in areas where rates of COVID-19 are low and where mask mandates have been removed, youth may be hesitant to stop wearing masks due to persistent fears and anxieties about getting sick [29].

Little is known about youth’s experiences with and concerns about Long Covid. Understanding youth perspectives on Long Covid may provide valuable insight into means of reducing the negative impact of the pandemic on this age group. In addition, because youth are at relatively lower risk of severe effects of acute COVID-19 infection, one of the main benefits of vaccination in this age group may be to protect against Long Covid [14]. Qualitative studies are especially powerful at eliciting the lived experiences of youth in their own words. Accordingly, this qualitative study sought to examine youth experiences with and perspectives on the long-term effects of COVID-19 infection.

## Methods

### Overall approach

We conducted a nationwide open-ended text message poll of youth in the United States aged 14–24 years to characterize their experiences with and concerns about Long Covid. Participants were included if they were able to receive and respond to text messages and could communicate in English. We then used thematic analysis to identify major themes from the qualitative text message responses.

### Data source

Participants in the poll came from the National MyVoice Text Message Cohort, a longitudinal mixed method study of youth [30, 31]. The MyVoice study aims to elicit youth’s perspectives to inform policies and practices that impact their wellbeing. Participants of the poll are sent weekly text message surveys with 3–5 questions each.

### Sample

Youth are recruited to enroll in MyVoice through social media advertisements. These advertisements are targeted with the goal of maximizing the degree to which the sample matches national demographic benchmarks for age, sex, race/ethnicity, and region of the country. These benchmarks are based on weighted samples of the American Community Survey [32]. Online consent is obtained from all youth, and demographic information is self-reported during enrollment via an online survey. [30] The demographic questions asked were standardized using other surveys of youth, including the Youth Risk Behavior Surveillance System and home affluence scales. [33, 34] This study was approved by the University of Michigan Institutional Review Board. Parental consent is waived for minors to ensure equitable enrollment and because the study was deemed of minimal risk. Participants were paid $1 per week for completing the weekly surveys.

### Survey

On April 23, 2021, participants received four open-ended questions regarding Long Covid via text message:

1. Have you or anyone you know tested positive for COVID-19?
2. What symptoms did you/they have? How long did the symptoms last?
3. Some people do not fully recover from COVID-19 weeks or even months after first experiencing symptoms. Have you or anyone you know experienced any long-term symptoms like this? Tell us about it.
4. Are you concerned about having long-term symptoms from COVID-19? Why or why not?

### Analysis

Three members of the study team conducted a thematic analysis of the text message responses using a content analysis. [35–37] For each poll question, two investigators reviewed all responses, iteratively identified thematic codes, and then a codebook was created for each poll question and refined through discussion among the authors. Three investigators then worked to manually apply the codes to responses for each question as per codebook, with two investigators fully coding each question independently. Any discrepancies in coding were discussed until consensus was reached between the coders. Prevalence of summary codes were summarized using descriptive statistics. Demographic information, including age, gender, race/ethnicity, education level, parental education level, free/reduced lunch status, and region were collected at study enrollment and summarized using descriptive statistics.

## Results

Out of 1,150 youth who received our question set, 991 submitted responses (86.2% response rate). Demographic characteristics of the sample are summarized in Table 1. Respondents were 19.3 years old on average (SD: 2.4), 49.0% male (n = 486), 62.6% non-Hispanic white (n = 620), and 37.4% (n = 368) qualified for free or reduced lunch, which are available to students of families below a certain income threshold and is used as a proxy for socioeconomic status in youth. Table 2 summarizes response rates for each survey question.

**Table 1:** Respondent demographic characteristics (n = 991)

Demographic characteristic	n (%)
**Age (n = 991)**	
14–17	254 (25.6)
18–24	737 (74.4)
**Gender (n = 991)**	
Male	486 (49.0)
Female	398 (40.2)
Other	107 (10.8)
**Race and Ethnicity (n = 990)**	
Non-Hispanic White	620 (62.6)
Non-Hispanic Black	64 (6.5)
Hispanic	103 (10.4)
Non-Hispanic Other	203 (20.5)
**Education level (n = 991)**	
Less than high school*	270 (27.3)
High school grad	142 (14.3)
Some college or tech school	401 (40.5)
Associate’s degree or tech grad	29 (2.9)
Bachelor’s degree or higher	149 (15.0)
**Region (n = 980)**	
Midwest	322 (32.9)
Northeast	176 (18.0)
South	268 (27.4)
West	214 (21.8)
**Received free or reduced lunch (n = 985)**	
Yes	368 (37.4)
No	617 (62.6)
*includes respondents still in high school	

**Table 2:** Questions, themes, and representative quotes

Question, Theme	n (%)	Representative quote
**Have you or anyone you know tested positive for COVID-19? (n = 987)**		
Yes	739 (75)	“Yes, my mom and my sister”
Me	48 (6)	“Yes, I tested positive 3 months ago”
Others	302 (41)	“I have not but some of my friends have”
Both me and others	23 (3)	“Yes me and multiple people I know have gotten covid”
Did not specify	366 (50)	“Yes”
No	248 (25)	“No one I know has tested positive for COVID-19”
**What symptoms did you/they have? How long did the symptoms last? (n = 948)**		
Symptoms	551 (58)	
Fever	235 (43)	“Fever, chills, loss of taste and smell…”
Cough	176 (32)	“They were experiencing cough and trouble breathing”
Loss of taste	176 (32)	“They lost their sense of smell and taste”
Loss of smell	156 (28)	“…Usually fatigue and loss of smell.”
Fatigue	151 (27)	“They were tired and had headaches”
Timeline	414 (44)	
A few days	85 (21)	“…Usually 3–7 days”
One week	120 (29)	“…lasted for about a week”
Two weeks	113 (27)	“The symptoms disappeared after 15 days”
Three weeks or more	124 (30)	“…I still have a cough that won’t go away”
**Some people do not fully recover from COVID-19 weeks or even months after first experiencing symptoms. Have you or anyone you know experienced any long-term symptoms like this? Tell us about it. (n = 914)**		
None	533 (58)	“No, I do not know someone who has persisted with the symptoms of covid.”
Yes	295 (32)	“Yes, a friend”
Loss of taste	99 (34)	“Yes my friend… she’s barely getting her taste back
Loss of smell	83 (28)	“Their sense of smell still hasn’t recovered fully”
Shortness of breath and/or lung damage	45 (15)	“Yes, my neighbors still have trouble breathing.”
Fatigue	39 (13)	“Tiredness has persisted”
Other health issues	83 (28)	“Yes, some still have brain fog” “Yes my sister has heart problems and stuff now”
Not Applicable or Not Sure	86 (9)	“N/A”, “I’m not sure”
**Are you concerned about having long-term symptoms from COVID-19? Why or why not?** **(n = 902)**		
Yes	450 (50)	
No	409 (45)	
Both Yes and No	43 (5)	“I am but now that I’ll be getting my second dose of the vaccine I’m not as concerned“
*Reasons worried*		
Generally Damaging	108 (12)	“A lot, I don’t want something bad to happen to me”
Long-term symptoms	91 (18)	“Yes, I don’t want anything affecting my health and I don’t want to have long lasting symptoms”
Fear of unknown	70 (14)	“I am concerned because we don’t know if it could cause severe effects later in life”
Quality of life/future	63 (13)	“I am concerned about the long term symptoms since they can impact your quality of life”
Preserve taste or smell	36 (7)	“yes. i would hate to have altered taste/smell or to have respiratory problems”
Underlying condition	34 (7)	“…I’m concerned about the long-term symptoms from Covid because I have underlying conditions…”
Affect on others	22 (2)	“Yes I would be concerned about it because I don’t want to get others around me sick”
Fear of Death	14 (3)	“I’m concerned about catching the virus and experiencing long-term effects or death.”
Concern about mental health	13 (3)	“…I cannot imagine the toll it must take on you mentally & emotionally if you never fully recover from COVID-19…”
*Reasons not worried*		
Vaccinated	133 (29)	“No because I got the vaccine”
Not had Covid	109 (24)	“No because I haven’t had covid”
Young/healthy	53 (12)	“No I’m young and pretty healthy”
Taking precautions	38 (8)	“No, because I am taking precautions to not get covid.”
Likely not severe	32 (7)	“No I’m not I feel like covid is no different than other sicknesses“
Already had Covid	25 (6)	“No I am not because I already had it and was asymptomatic“

The majority of youth (75%; n = 739 out of 987 respondents to this question) surveyed either had COVID-19 themselves or knew someone who did. Of these respondents, 6% (n = 48) specified they had COVID themselves and 41% (n = 302) knew a friend or relative with COVID; 23 respondents (3%) indicated that both themselves and someone they knew had COVID.

Regarding symptoms from COVID-19 infection, the most commonly referenced were fever (43%; n = 235 out of 948 respondents to this question), cough (32%; n = 176), and a loss of taste (32%; n = 176). Loss of smell (28%; n = 156) and fatigue (27%; n = 151) were also commonly noted. Nearly half of respondents (44%; n = 414) included a duration of these symptoms. Of these responses, 30% (n = 124) indicated that these symptoms lasted more than three weeks or were ongoing (“I still have a cough that won’t go away”). Other individuals reported that these symptoms disappeared after just a few days (21%, n = 85), one week (279%, n = 120), or two weeks (27%, n = 113).

Approximately one third (32%; n = 295 out of 914 respondents to this question) of youth indicated that they knew someone who had suffered from Long Covid (“Yes, I have a friend who still suffers from chest pain and running out of breath quickly”). Of these responses, the most common long-term symptoms were loss of taste (34%; n = 99 “…she’s barely getting her taste back”) and loss of smell (28%; n = 83 “their sense of smell hasn’t recovered fully”). Other commonly reported long-term symptoms included shortness of breath or lung issues (15%; n = 45 “my neighbors still have trouble breathing”), fatigue (13%; n = 39 “tiredness has persisted), and headache (6%; n = 18` “my mother in law is still having headaches”).

When asked whether they themselves were concerned about having long-term symptoms of COVID-19, half of all respondents to this question (50%; n = 450 out of 902) reported that they were concerned, 45% (n = 409) reported that they were not concerned, and 5% (n = 43)answered that they were concerned in some aspects, but not in others. Although respondents may report for both youth and adults, these numbers are comparable to prior data on Long Covid in youth. Reasons that individuals reported concern about Long Covid were the general damage the virus could cause (22%; n = 108 “Because some people’s organs dont work well anymore. That is a lifetime issue”), ongoing bothersome symptoms (18%; n = 91 “Yes, I’ve heard of people having lasting symptoms that never go away”), the unknown impact COVID could have on their health (14%; n = 70 “since not all the long term effects are known and permanent damage to the human body is a concern.”), the need to preserve taste or smell (7%; n = 36 “I would miss being able to taste so many of my favorite foods”), and underlying health conditions (7%; n = 34 “I’m worried about long term lung and breathing symptoms because of my asthma.”). Reasons individuals were not concerned about Long Covid included receiving the vaccine (29%; n = 133 “No because I’m fully vaccinated”), not contracting COVID yet (24%; n = 109 “No. I’ve never had COVID and I don’t go enough places to get it”), being young and healthy (12%; n = 53 “I’m very young, otherwise healthy, and have a very effective immune system “), and taking precautions (8%; n = 38 “No because I protect myself by following guidelines”).

## Discussion

A significant majority of the nearly 1,000 youth who responded to our nationwide text message survey reported that they either tested positive for COVID or knew someone who did. The numbers have undoubtedly increased since this survey was released in April 2021, due to the continuation of the pandemic, evolution of variants [38], relaxation of COVID-19 restrictions [39], and an end to the COVID- 19 national emergency. Although youth have suffered less illness and death from COVID compared to adults [40], our results highlight that they have experienced unique and significant impacts from the pandemic, and will continue to face negative repercussions due to Long Covid. This necessitates a concerted effort to better understand and alleviate the physical, psychological, and social impacts of Long Covid on our youth.

32% of the respondents who included a time frame (n = 295) reported that they or someone they knew had Long Covid symptoms. Although our respondents may be reporting a time frame from both youth and adults in their lives, this time frame is comparable to prior data in youth which found the prevalence of Long Covid variable, between 4 to 66% [14]. According to a meta-analysis of both controlled and uncontrolled studies on persistent COVID-19 symptoms among children and youth, 6.7% of symptomatic cases experienced on-going symptoms [41]. The difference between the prevalence of persistent symptoms reported in our sample and this meta-analysis is likely due to the fact that our study included individuals up to age 24, and youth in our study reported not only their own symptoms, but also those of people they knew that had COVID, whose ages were not specified. Furthermore, 16% of respondents reported fatigue as a symptom, which may be attributable to either COVID-19 infection or to social and emotional impacts of the pandemic. In addition, respondents may conflate fatigue due to other causes with Long Covid induced fatigue. Regardless of the precise prevalence of Long Covid among youth, the fact that many youth in our study reported persistent symptoms of their own, and 50% reported concern about having long-term symptoms from COVID-19, indicates a societal need to support youth. Healthcare providers, teachers, parents, and employers, for example, must be equipped to screen, counsel, and accommodate youth who either fear or experience long-term symptoms from COVID-19, or who must support family members experiencing Long Covid.

The most commonly reported long-term symptoms of COVID-19 which youth in our survey perceived were loss of taste, loss of smell, respiratory symptoms, and fatigue. Other notable persistent symptoms reported included headache, brain fog, and body aches. These symptoms are consistent with previous studies on Long Covid among youth [41], which showed that the most common reported symptoms across studies were headache, fatigue, sleep disturbance, concentration difficulties, abdominal pain, myalgia or arthralgia, congestion, cough, chest tightness or pain, loss of appetite or weight, anosmia, and rash [14, 41]. The Long Covid symptoms reported among youth in our sample are also similar to those reported among adults [6], a finding that is consistent with the fact that our sample included individuals up to 24 years of age. Among adults, these symptoms have resulted in functional limitations and loss of productivity and resources [42]. For example, two-thirds of adults with Long Covid say that they are unable to perform usual duties and activities [42, 43]. Youth in our study have reported similar symptoms personally, or in those they know with persistent COVID-19 symptoms, suggesting that their typical daily activities may also be compromised. The debilitation caused by these symptoms may be especially detrimental among youth, whose development and learning is ongoing and often must occur in a sequential fashion. Long Covid, coupled with the disruption caused by virtual schooling and decreased social interaction, is likely to have a long-term detrimental impact on youth.

While 32% of all respondents experienced, or knew someone who experienced Long Covid, a much larger percentage of respondents (50%) expressed some degree of concern about the long-term symptoms COVID-19. This level of concern among youth, especially during a period of growth and turbulence in their lives, has serious implications for medical professionals, policy makers, and those invested in the future health of youth. Physicians, nurses, and other medical professionals will be at the forefront of managing the impact of Long Covid on youth. While the physical effects of Covid on youth are substantial, the worry over Long Covid could go unnoticed, leading to poor health outcomes. Our study emphasizes the importance of preparing clinical providers to address these concerns with youth, and to develop approaches for broaching this subject during clinical visits. Medical organizations should put forth recommendations for providers to effectively screen for and manage the long-term sequelae of Covid among youth. At the policy level, these findings should prompt increased funding to better understand how Long Covid affects youth and fund programs designed to mitigate these impacts.

Greater consumption of COVID-19-related news coverage has been correlated with increased anxiety and depression symptoms among youth during the pandemic. [26, 29, 44, 45]. However, awareness of COVID-19 prevention and control measures and awareness of COVID-19 trends among youth have also been identified as protective factors against depressive and anxiety symptoms [46]. These findings suggest that while consuming media about Long Covid generally may exacerbate concerns and anxieties among youth, informing youth about particular aspects of Long Covid, such as the epidemiology of the condition and Long Covid prevention tactics, may both inform youth and help alleviate those concerns. Our study supports the idea that reducing the uncertainty about Long Covid among youth may decrease anxieties surrounding the condition, as 14% of respondents cited uncertainty about Long Covid as one of the reasons that they were worried. Social marketing campaigns designed for youth have successfully engaged youth around health promotion [47, 48], and youth participation in content creation for these campaigns may contribute to their effectiveness [49]. Our study suggests that there is a need for youth-centered public health communication about the emerging evidence behind Long Covid management and prevention strategies.

Many youth who were not concerned about the effects of Long Covid cited the vaccine and COVID precautions as protective factors. This finding suggests that vaccine promotion messaging may resonate in this age group if it focuses on the impact of vaccination on Long Covid. This correlates with other surveys of youth, which found that 75.9% were willing to receive the vaccine to a degree [50]. Some youth in our study (6%) reported that they were not concerned about Long Covid because they are young and healthy, indicating there remains some youth who perceive COVID-19 to be a virus that does not affect themselves or their peers. It is important to highlight that 12% of respondents cited never being infected with COVID-19 as their primary reason for not being concerned about Long Covid. Inevitably, more youth became infected with COVID-19 after responding to our survey and more youth may experience first-hand the uncertainty surrounding symptoms that persist beyond acute infection. This trend may lead to increased mental health burden associated with the long-term effects of COVID.

### Limitations

The results of this study should be interpreted with several factors in mind. Although we had a large number of respondents, our sample is not nationally representative, which may limit the ability to extrapolate our data. In addition, these responses were collected in April of 2021. In the ever-changing context of the COVID-19 pandemic, it is highly probable that some of the perceptions we discussed have shifted. Finally, the survey questions were written to provide information to youth about Long Covid while also eliciting their perspectives, which could cause an overreporting of symptoms. However, there is still great value in understanding youth concerns about Long Covid, as these concerns may have increased over time. It is also possible that those with persistent symptoms were more likely to respond to the text message questions than those unaffected by COVID-19 or Long Covid, but the study’s high response rate likely helped mitigate any selection bias in our results.

## Conclusion

The findings of our survey indicate that nearly one third of respondents experienced or knew someone who experienced Long Covid. Many youth (50%) are concerned about Long Covid even if they, or someone they knew, did not have Long Covid. Reasons for this concern included the general damage the virus could cause, potential for bothersome symptoms, the uncertainty surrounding the virus’ impact, the need to preserve taste or smell, and underlying health conditions. Reasons individuals were not concerned about Long Covid included receiving the vaccine, not yet contracting COVID-19, being young and healthy, and taking precautions. Our findings also suggest that vaccination messaging that focuses on Long Covid prevention may be especially effective. Furthermore youth-centered public health communications that keep youth informed about the emerging evidence behind Long Covid management and prevention may not only reduce COVID-19 transmission, but also alleviate worries and concerns about Long Covid among youth.

## Data Availability

The datasets used and/or analyzed during the current study are available from the corresponding author on reasonable request.

## References

[1] Centers for Disease Control and Prevention. COVID data tracker Available from: https://covid.cdc.gov/covid-data-tracker/#cases_casesper100klast7days.

[2] Carfi A, Bernabei R, Landi F, Gemelli Against, C-P-ACSG (2020). Persistent symptoms in patients after acute COVID-19. JAMA.

[3] Garrigues E, Janvier P, Kherabi Y, Le Bot A, Hamon A, Gouze H (2020). Post-discharge persistent symptoms and health-related quality of life after hospitalization for COVID-19. J Infect.

[4] Petersen MS, Kristiansen MF, Hanusson KD, Danielsen ME, á Steig B, Gaini S (2021). Long COVID in the Faroe Islands: a longitudinal study among nonhospitalized patients. Clin Infect Dis.

[5] Callard F, Perego E (2021). How and why patients made long Covid. Soc Sci Med.

[6] Bliddal S, Banasik K, Pedersen OB, Nissen J, Cantwell L, Schwinn M (2021). Acute and persistent symptoms in non-hospitalized PCR-confirmed COVID-19 patients. Sci Rep.

[7] Mandal S, Barnett J, Brill SE, Brown JS, Denneny EK, Hare SS (2021). Long-COVID’: a cross-sectional study of persisting symptoms, biomarker and imaging abnormalities following hospitalisation for COVID-19. Thorax.

[8] Nalbandian A, Sehgal K, Gupta A, Madhavan MV, McGroder C, Stevens JS (2021). Post-acute COVID-19 syndrome. Nat Med.

[9] Alwan NA, Johnson L (2021). Defining long COVID: going back to the start. Med.

[10] Buonsenso D, Munblit D, De Rose C, Sinatti D, Ricchiuto A, Carfi A (2021). Preliminary evidence on long COVID in children. Acta Paediatr.

[11] Huang L, Yao Q, Gu X, Wang Q, Ren L, Wang Y (2021). 1-year outcomes in hospital survivors with COVID-19: a longitudinal cohort study. Lancet.

[12] Huang Y, Pinto MD, Borelli JL, Mehrabadi MA, Abrihim H, Dutt N (2021). COVID symptoms, symptom clusters, and predictors for becoming a long-hauler: looking for clarity in the haze of the pandemic. medRxiv.

[13] Goërtz YM, Van Herck M, Delbressine JM, Vaes AW, Meys R, Machado FV et al. Persistent symptoms 3 months after a SARS-CoV-2 infection: the post-COVID-19 syndrome? ERJ Open Research. 2020;6(4). 10.1183/23120541.00542-2020 PMC7491255 33257910

[14] Zimmermann P, Pittet LF, Curtis N (2021). How common is long COVID in children and adolescents?. Pediatr Infect Dis J.

[15] Lopez-Leon S, Wegman-Ostrosky T, Ayuzo Del Valle NC, Perelman C, Sepulveda R, Rebolledo PA (2022). Long-COVID in children and adolescents: a systematic review and meta-analyses. Sci Rep.

[16] Noval Rivas M, Porritt RA, Cheng MH, Bahar I, Arditi M (2022). Multisystem inflammatory syndrome in children and long COVID: the SARS-CoV-2 viral Superantigen hypothesis. Front Immunol.

[17] Gupta M, Gupta N, Esang M. Long COVID in children and adolescents. Prim Care Companion CNS Disord. 2022;24(2). 10.4088/PCC.21r03218. 10.4088/PCC.21r03218 35486940

[18] Ahmed SP, Bittencourt-Hewitt A, Sebastian CL (2015). Neurocognitive bases of emotion regulation development in adolescence. Dev Cogn Neurosci.

[19] Spear L. The behavioral neuroscience of adolescence. WW Norton & Company; 2010.

[20] Lee FS, Heimer H, Giedd JN, Lein ES, Sestan N, Weinberger DR (2014). Mental health. Adolescent mental health–opportunity and obligation. Science.

[21] Samji H, Wu J, Ladak A, Vossen C, Stewart E, Dove N (2021). Mental health impacts of the COVID-19 pandemic on children and youth–a systematic review. Child Adolesc Ment Health.

[22] McElroy E, Patalay P, Moltrecht B, Shevlin M, Shum A, Creswell C (2020). Demographic and health factors associated with pandemic anxiety in the context of COVID-19. Br J Health Psychol.

[23] Amran MS (2020). Psychosocial risk factors associated with mental health of adolescents amidst the COVID-19 pandemic outbreak.

[24] Cusinato M, Iannattone S, Spoto A, Poli M, Moretti C, Gatta M (2020). Stress, resilience, and well-being in italian children and their parents during the COVID-19 pandemic. Int J Environ Res Public Health.

[25] Scott SR, Rivera KM, Rushing E, Manczak EM, Rozek CS, Doom JR (2021). I hate this: a qualitative analysis of adolescents’ self-reported challenges during the COVID-19 pandemic. J Adolesc Health.

[26] Ellis WE, Dumas TM, Forbes LM (2020). Physically isolated but socially connected: psychological adjustment and stress among adolescents during the initial COVID-19 crisis. Can J Behav Sci.

[27] Murata S, Rezeppa T, Thoma B, Marengo L, Krancevich K, Chiyka E (2021). The psychiatric sequelae of the COVID-19 pandemic in adolescents, adults, and health care workers. Depress Anxiety.

[28] Zhang L, Zhang D, Fang J, Wan Y, Tao F, Sun Y (2020). Assessment of mental health of chinese primary school students before and after school closing and opening during the COVID-19 pandemic. JAMA Netw Open.

[29] New York Times. For some teens, as masks come off, anxiety sets in Available from: https://www.nytimes.com/2022/03/17/well/family/teenage-student-mask-anxiety.html.

[30] DeJonckheere M, Nichols LP, Moniz MH, Sonneville KR, Vydiswaran VGV, Zhao X (2017). MyVoice national text message survey of youth aged 14 to 24 years: study protocol. JMIR Res Protoc.

[31] MyVoice. Transparency Initiative Available from: https://hearmyvoicenow.org/research/transparency/.

[32] United States Census Bureau. American Community Survey (ACS) Available from: https://www.census.gov/programs-surveys/acs.

[33] Centers for Disease Control and Prevention. Adolescent and school health: YRBSS Questionnaires Available from: https://www.cdc.gov/healthyyouth/data/yrbs/questionnaires.htm.

[34] Wardle J, Robb K, Johnson F (2002). Assessing socioeconomic status in adolescents: the validity of a home affluence scale. J Epidemiol Community Health.

[35] Forman J, Damschroder L. Qualitative content analysis. Empirical methods for bioethics: a primer. Emerald Group Publishing Limited; 2007. pp. 39–62.

[36] Hsieh HF, Shannon SE (2005). Three approaches to qualitative content analysis. Qual Health Res.

[37] Thompson Burdine J, Thorne S, Sandhu G (2021). Interpretive description: a flexible qualitative methodology for medical education research. Med Educ.

[38] van Oosterhout C, Hall N, Ly H, Tyler KM. COVID-19 evolution during the pandemic–implications of new SARS-CoV-2 variants on disease control and public health policies. Taylor & Francis; 2021. pp. 507–8. 10.1080/21505594.2021.1877066 PMC7849743 33494661

[39] Sheikh A, Sheikh A, Sheikh Z, Dhami S. Reopening schools after the COVID-19 lockdown. J Global Health. 2020;10(1). 10.7189/jogh.10.010376 PMC7321012 32612815

[40] Bhattacharyya A, Seth A, Srivastava N, Imeokparia M, Rai S. Coronavirus (COVID-19): a systematic review and meta-analysis to evaluate the significance of demographics and comorbidities. Res Square. 2021.

[41] Zavala M, Ireland G, Amin-Chowdhury Z, Ramsay ME, Ladhani SN (2022). Acute and persistent symptoms in children with PCR-confirmed SARS-CoV-2 infection compared with test-negative children in England: active, prospective, national surveillance. Clin Infect Dis.

[42] Alwan NA (2021). The road to addressing long COVID. Science.

[43] Norrefalk JR, Borg K, Bileviciute-Ljungar I (2021). Self-scored impairments in functioning and disability in post-COVID syndrome following mild COVID-19 infection. J Rehabil Med.

[44] Duan L, Shao X, Wang Y, Huang Y, Miao J, Yang X (2020). An investigation of mental health status of children and adolescents in china during the outbreak of COVID-19. J Affect Disord.

[45] Kiss O, Alzueta E, Yuksel D, Pohl KM, de Zambotti M, Műller-Oehring EM (2022). The pandemic’s toll on young adolescents: prevention and intervention targets to preserve their mental health. J Adolesc Health.

[46] Zhou SJ, Zhang LG, Wang LL, Guo ZC, Wang JQ, Chen JC (2020). Prevalence and socio-demographic correlates of psychological health problems in chinese adolescents during the outbreak of COVID-19. Eur Child Adolesc Psychiatry.

[47] Cowell AJ, Farrelly MC, Chou R, Vallone DM (2009). Assessing the impact of the national ‘truth’ antismoking campaign on beliefs, attitudes, and intent to smoke by race/ethnicity. Ethn Health.

[48] Davis KC, Farrelly MC, Messeri P, Duke J (2009). The impact of national smoking prevention campaigns on tobacco-related beliefs, intentions to smoke and smoking initiation: results from a longitudinal survey of youth in the United States. Int J Environ Res Public Health.

[49] Gupta RS, Lau CH, Warren CM, Lelchuk A, Alencar A, Springston EE (2013). The impact of student-directed videos on community asthma knowledge. J Community Health.

[50] Brandt EJ, Rosenberg J, Waselewski ME, Amaro X, Wasag J, Chang T (2021). National Study of Youth opinions on vaccination for COVID-19 in the U.S. J Adolesc Health.

